# Isolation of mesenchymal stem cells from equine umbilical cord blood

**DOI:** 10.1186/1472-6750-7-26

**Published:** 2007-05-30

**Authors:** Thomas G Koch, Tammy Heerkens, Preben D Thomsen, Dean H Betts

**Affiliations:** 1 Department of Biomedical Sciences, University of Guelph, Guelph, ON, N1G 2W1, Canada; 2 Department of Basic Animal and Veterinary Sciences, University of Copenhagen, Grønnegårdsvej 7,1870 Frederiksberg C, Denmark

## Abstract

**Background:**

There are no published studies on stem cells from equine cord blood although commercial storage of equine cord blood for future autologous stem cell transplantations is available. Mesenchymal stem cells (MSC) have been isolated from fresh umbilical cord blood of humans collected non-invasively at the time of birth and from sheep cord blood collected invasively by a surgical intrauterine approach. Mesenchymal stem cells isolation percentage from frozen-thawed human cord blood is low and the future isolation percentage of MSCs from cryopreserved equine cord blood is therefore expectedly low. The hypothesis of this study was that equine MSCs could be isolated from fresh whole equine cord blood.

**Results:**

Cord blood was collected from 7 foals immediately after foaling. The mononuclear cell fraction was isolated by Ficoll density centrifugation and cultured in a DMEM low glucose based media at 38.5°C in humidified atmosphere containing 5% CO_2_. In 4 out of 7 samples colonies with MSC morphology were observed. Cellular morphology varied between monolayers of elongated spindle-shaped cells to layered cell clusters of cuboidal cells with shorter cytoplasmic extensions. Positive Alizarin Red and von Kossa staining as well as significant calcium deposition and alkaline phosphatase activity confirmed osteogenesis. Histology and positive Safranin O staining of matrix glycosaminoglycans illustrated chondrogenesis. Oil Red O staining of lipid droplets confirmed adipogenesis.

**Conclusion:**

We here report, for the first time, the isolation of mesenchymal-like stem cells from fresh equine cord blood and their differentiation into osteocytes, chondrocytes and adipocytes. This novel isolation of equine cord blood MSCs and their preliminary *in vitro *differentiation positions the horse as the ideal pre-clinical animal model for proof-of-principle studies of cord blood derived MSCs.

## Background

In 2004, 115 thousand new Thoroughbred foals were registered worldwide [1] for whom orthopaedic injuries will be the most common cause of lost training days or premature retirement [2-5]. Traumatic osteoarthritis (OA) is estimated to constitute 60% of all equine lameness issues making it the leading cause of equine lameness [6]. Osteochondrosis (OC) lesions are other important causes of joint disease in the horse [7,8].

Currently, there are no medical treatments available that can reverse cartilage injuries and most are only palliative in mild to moderate cases. Surgical cartilage and osteochondral resurfacing techniques in horses with focal OA or OC aims at either stimulated endogenous repair and or grafting of tissues [6]. Currently the surgical technique of choice for stimulated endogenous repair is abrasion arthroplasty alone or combined with subchondral bone microfracture [6]. The long-term clinical efficacy of this approach has not been reported. Grafting of equine tissues and cells for OA and OC repair remains experimental due to technical and biological problems. Retaining the transplant while maintaining articular surface congruity at the recipient site is a significant problem and the harvest of autologous graft material requires multiple surgeries or multiple surgery sites with increased risk to the patient [6,9]. Equine bone marrow derived autologous MSCs require time consuming *in vitro *propagation prior to transplantation and a decreased yield of stem cells from the bone marrow with increasing age makes the technique age-dependent which is paradoxical to the fact that many clinical cases are aged individuals [10]. Genetic modification of bone marrow derived equine MSCs might help to overcome some of these problems [11].

Mesenchymal stem cells or unrestricted somatic stem cells (USSCs) have been isolated from fresh umbilical cord blood of humans collected non-invasively at the time of birth [12,13] and from sheep cord blood collected invasively by a surgical intrauterine approach [14]. Human cord blood non-hematopoietic stem cells have been differentiated into multiple cell types such as endothelial cells, neurons, smooth muscle cells, adipocytes, chondroblasts and osteoblasts [12,13,15-23]. Cartilaginous tissue has been produced *in vitro *from sheep cord blood [14]. Comparative studies of human bone marrow, adipose tissue and cord blood derived MSCs revealed differences in isolation frequency, proliferative capacity, *in vitro *differentiation potential, telomere length, and telomerase activity [12,13,16]. Human cord blood derived hematopoietic stem cells might even be immunologically privileged compared to bone marrow derived hematopoietic stem cells [24].

In the horse, the isolation percentage of MSCs from peripheral blood is very low and bone marrow is currently the main source of equine MSCs [25-29]. There are no peer-reviewed reports of equine adipose tissue derived stem cells although commercial marketing of so-called equine adipose tissue derived stem cell therapy is taken place today [30]. There appears to be no published studies on equine cord blood stem cells, hematopoietic or mesenchymal, although commercial storage of equine cord blood for possible future autologous stem cell transplantations is available [29]. Mesenchymal stem cells isolation from thawed human cord blood is associated with significantly reduce success [31,32] and the future isolation percentage of MSCs from cryopreserved equine cord blood is therefore expectedly low.

Isolation of MSCs cells from equine umbilical cord blood could provide a non-invasive source of stem cells with potentially superior cellular characteristic to other equine stem cells with regard to immune tolerance, proliferative potential and differentiation potency. This study reports the isolation of cryopreservation and thawing tolerant MSCs from fresh equine cord blood with *in vitro *differentiation potential towards the osteogenic, chondrogenic and adipogenic cell lineages.

## Results

### Cord blood collection

No complications were encountered upon umbilical cord blood collection. The storage and transport temperature was 15–22°C (mean 17.9°C), transport time was 8–24 hours (mean 15 hours), sample volume was 65–250 ml (mean 168 ml ± 36.08 S.E. ml), and no sample had signs of coagulation or hemolysis.

### Isolation and propagation

**Figure 1:**
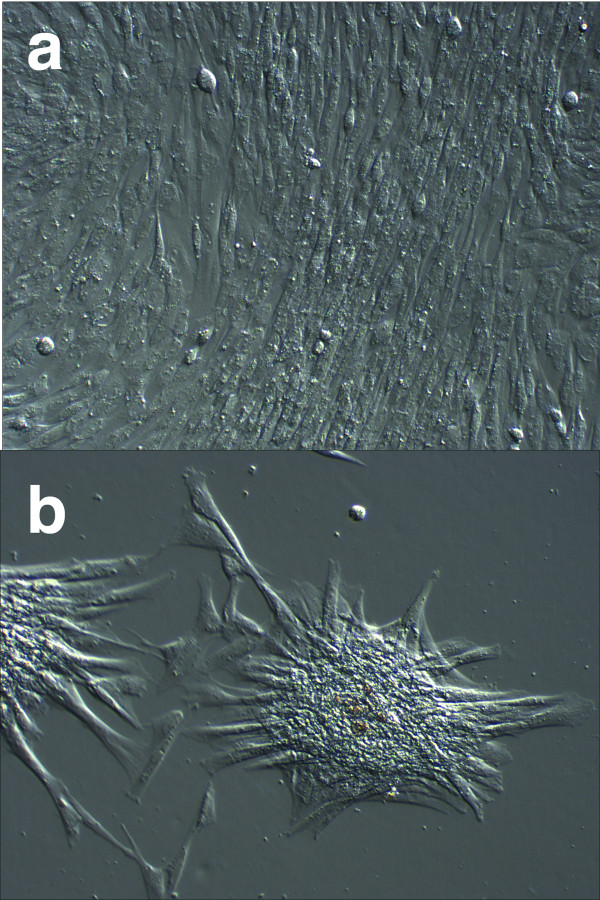
**Isolation of mesenchymal stem cells from equine umbilical cord blood**. **(a) **Monolayer of rapidly expanding adherent spindle-shaped fibroblastoid cells compatible with undifferentiated mesenchymal stem cells (× 100). **(b) **Three-dimensional, relief contrast image of cell cluster of rapidly expanding adherent spindle-shaped fibroblastoid cells compatible with undifferentiated mesenchymal stem cell morphology (× 100).

In 4 out of 7 cord blood samples colonies with the classical MSCs morphology of adherent fibroblastoid spindle-shaped cells growing in a monolayer were observed (Fig. 1a) giving an isolation percentage success rate of 4/7 (× 100 = 57%). The numbers of primary colonies from each sample were 1, 5, 2 and 1, respectively. Colonies were observed as early as 3 days post seeding and the first subculture was done 7 days after initial seeding. One sample containing putative MSCs colonies were subsequently discarded due to fungal contamination. Although the over-all appearance of passaged cells was that of spindle-shaped cells, cellular morphology did vary between very slender and elongated to more cuboidal cells with shorter cytoplasmic extensions with a layered growth (Fig. 1b). These morphological differences were seen within and between cord blood samples. Undifferentiated cells have been passaged up to 10 times and population-doubling times were calculated for a subset of cell passages. The population doubling time varied between 0.49 and 1.22 per day with a mean of 0.77.

#### *In vitro *differentiation results

All the depicted results in figures and pictures of the *in vitro *differentiation studies are from experiments performed on cryopreserved and thawed cells. Similar results were obtained from experiments performed with "fresh" undifferentiated cells which had not been previously cryopreserved.

**Figure 2:**
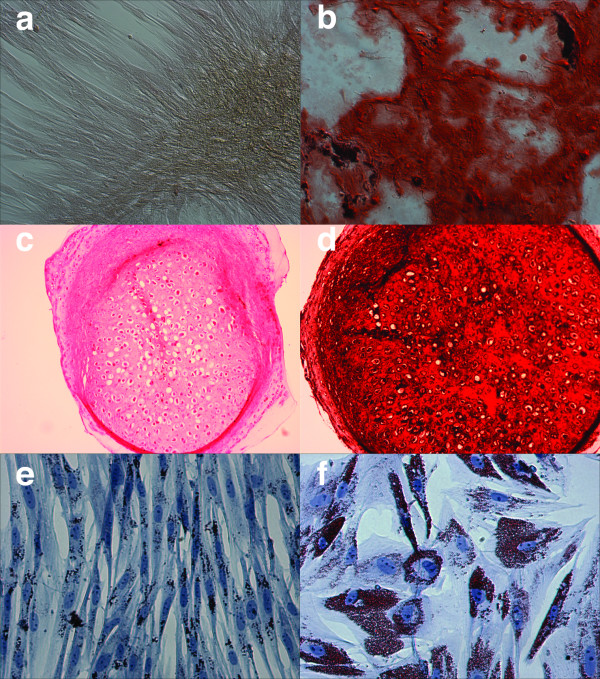
***In vitro *differentiation studies**. **(a) **Osteogenic control after 21 days in regular expansion media maintained normal morphology and stain negative for Alizarin Red S (× 100). **(b) **Osteogenic induction after 21 days shows marked morphological changes and extensive extra cellular calcium deposition as demonstrated by positive Alizarin red S staining (× 100). **(c) **Haematoxylin and Eosin stain of chondrogenic induced micro pellet showing classical hyaline cartilage morphology with lacunae containing chondrocytes surrounded by extra cellular matrix (× 100). **(d) **Safranin O staining of glycosaminoglycans in the cartilage matrix (× 100). **(e) **Adipogenic control culture after exposure to regular expansion media shows maintenance of undifferentiated morphology and minor lipid droplet deposition (× 200). **(f) **Positive adipogenic induction after culture in adipogenic induction media spiked with 15% rabbit serum as demonstrated by morphological change towards larger cells with marked lipid droplet deposition stained with Oil Red O (× 200).

**Figure 3:**
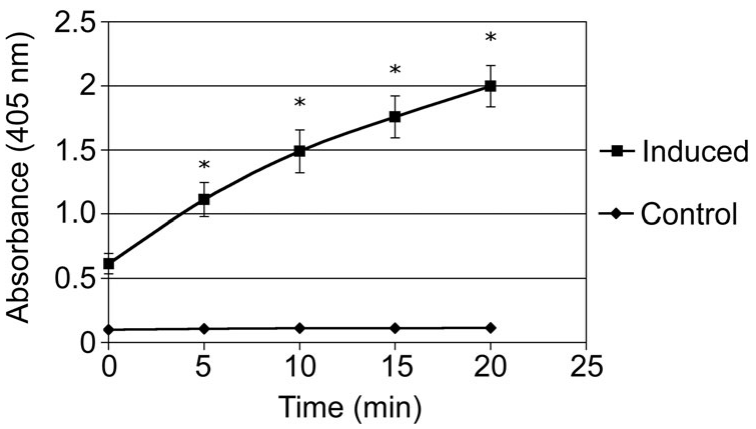
**Alkaline phosphatase activity in control and induced osteogenic differentiation samples**. Significant statistical difference (*) at the 95% level between control and induced was found at 5, 10, 15 and 20 minutes with p-values of < 0.001 at each time point. No difference was present at time 0 (p = 0.1418).

**Figure 4:**
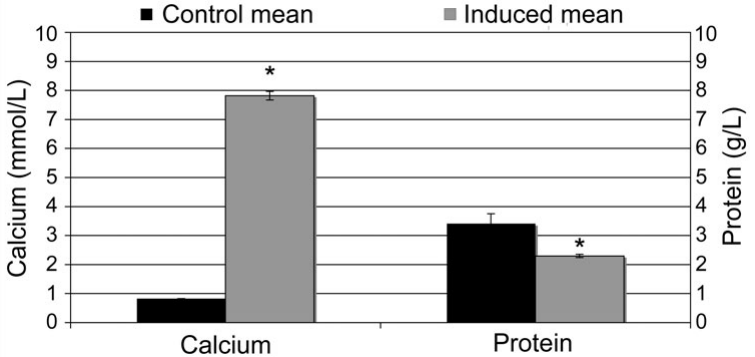
**Mean calcium and protein levels in osteogenic differentiation studies of cord blood mesenchymal stem cells**. Significant difference in calcium deposition between control and osteo-induced cultures was apparent (*, p < 0.001). There were significantly elevated protein levels per culture well in controls compared to osteo-induced cultures (*, p < 0.001).

Osteogenic induced cell cultures changed morphology from adherent monolayer of swirling spindle-shaped cells, which was still apparent in the control cultures (Fig. 2a), to layered cell clusters surrounded by a matrix-like substance positive upon Alizarin Red S (Fig. 2b) and von Kossa staining (data not shown). Statistically significant (p < 0.001) higher quantities of calcium deposition and alkaline phosphatase activity at the 95% confidence level were also demonstrated in these osteogenic induced culture wells (Fig. 3 &4). The total protein content of the culture wells was significantly (p < 0.001) lower in the osteogenic induced wells compared to the control wells indicating a lower cellularity in the induced wells (Fig. 4). Classical histological morphology of hyaline cartilage after 2 and 4 weeks of culture was evident upon staining of pellet sections with hematoxylin and eosin (Fig. 2c). Safranin O staining revealed marked deposition of glycosaminoglycans in the matrix (Fig. 2d). In comparison with control cultures in regular expansion media (Fig. 2e), marked adipogenic differentiation occurred in the presence of adipogenic induction media spiked with rabbit serum (Fig. 2f).

## Discussion

Equine umbilical cord blood was collected without complications to the mare or foal at the time of foaling using a standard commercially available blood transfusion bag system. Mesenchymal stem cells with *in vitro *differentiation potency towards the osteogenic, chondrogenic and adipogenic cell fates were successfully isolated from the cord blood. The undifferentiated MSCs have been cryopreserved, thawed and expanded post-thawing without obvious loss of morphology, proliferative potential and differentiation capacity.

The volume of blood collected was markedly higher (mean 168 ml) than what has been reported in human studies (mean 42.8 ml, range 13–80 ml [12]; mean 60.9 ml, range 17–141,5 ml [33]). The MSCs isolation frequency of 57% is very similar to reported isolation frequencies in human studies of up to 63% [12]. However, these high isolation frequencies are often only obtained in a subset of human cord blood samples after applying various critical parameters [12]. Correlating the number of primary colonies to the volume of blood processed might help determine whether this apparently high success rate is due to the higher volume of equine blood collected or due to higher inherent precursor frequency of these cells in equine cord blood compared to human cord blood. Here, the precursor frequency was very low with primary colonies varying between 1 and 5 per sample, indicating that this stem cell population is a very rare cell type, which is in correlation with human studies [12,13]. The different morphologies of undifferentiated cells are intriguing since they might reflect inherently different stem cell phenotypes. Morphological and differentiation potency differences between USSCs and "true" MSCs have been proposed [32]. Mesenchymal stem cells apparently form Alizarin Red positive nodules after six to eight passages and lack endodermic potential; whereas USSCs retain spindle-shaped fibroblastoid cell morphology in monolayer and have endodermic potential [13,32]. Whether the different morphologies noted in this study reflect USSCs and MSCs is undetermined, but warrants further investigation. So-far reduced growth rates have not been observed during sub-culturing. However, the number of samples is insufficient to draw any conclusion regarding the proliferative potential of these cells and how their proliferative potential might compare to equine bone marrow, and possibly equine adipose-derived MSCs.

Although further molecular characterization is required, the osteogenic and chondrogenic potency of these cells has been convincingly demonstrated in this study. No monoclonal antibodies are presently available for immunophenotyping equine mesenchymal stem cells and a specific panel of MSC markers has not been reported in the scientific literature. If and when such an equine MSC marker panel is available, screening and cell separation of the undifferentiated umbilical cord blood derived MSCs would be very interesting in order to compare the immunophenotye profile of these cells with that of human cord blood derived MSCs and that of equine bone marrow and possibly adipose tissue derived MSCs. Nevertheless, osteogenesis has been demonstrated by both morphological and functional criteria as established in previous studies of MSCs [12,13]. Chondrogenic assessment was used using established methods and our histological findings of typical hyaline cartilage morphology with glycosaminoglycan containing matrix is in accordance with other similar studies of MSCs [13,34]. Koerner and colleagues (2006) reported that adult equine peripheral blood progenitor cells did not show any capacity to produce cartilage at the histological level [25]. This apparent difference in chondrogenic differentiation potential between equine cord blood and adult peripheral blood is undetermined but warrants further investigation. Adipogenesis of peripheral blood derived MSCs from adult horses was previously shown successfully, although reduced adipogenic potential was noted compared to human MSCs [25]. Here, rabbit serum was added to the commercial adipogenic induction media based on the work by Janderova and colleagues (2003) in which superior adipogenesis was demonstrated [35]. Adipogenic differentiation of the equine cord blood derived MSCs were only successful with the addition of rabbit serum to the media. Conclusions to the adipogenic potential of adult equine peripheral blood MSCs versus equine cord blood MSCs cannot be made at this point in time, since the adipogenic potential observed for both cell types was demonstrated under different culture conditions. Whether there is an inherent difference between these blood-derived blood MSCs responsible for our failure to induce adipogenesis without the addition of rabbit serum is undetermined. Also, the effect of rabbit serum on the adipogenic differentiation potential of adult equine blood MSCs is unknown. Adipogenesis was unsuccessful for human cord blood derived MSCs cultured in rabbit serum deficient media [16]. Whether this is a methodological difference or a cellular difference between equine and human cord blood MSCs warrants further studies.

Equine cord blood MSCs might have superior cellular characteristic to other equine stem cells with regard to immune tolerance, proliferative potential and differentiation potency, which are desirable traits in selection of cells for tissue engineering. Comparative studies of human bone marrow, adipose tissue and cord blood derived MSCs revealed lower isolation frequency from cord blood compared to bone marrow and adipose tissue, but cord blood derived MSCs had higher proliferative potential than both bone marrow and adipose tissue derived MSCs [16]. Longer telomeres, higher telomerase activity and superior *in vitro *proliferation capacity and differentiation potential of human cord blood MSCs compared to human bone marrow and adipose tissue derived MSCs have also been reported [12,13]. Human leukemia patients transfused with HLA mismatched cord blood compared to transfusions of HLA matched bone marrow showed no difference in treatment outcome [24] indicating that cord blood derived stem cells might be immunologically privileged compared to bone marrow derived stem cells. The exact immunogenic potential of umbilical cord blood derived MSCs remain undetermined. Banking of ELA matched cord blood derived stem cells would therefore be the safest approach for allogenic-based tissue engineering or stem cell-based therapies at the moment. Isolation of MSCs from cryopreserved cord blood is very difficult and MSCs isolation and expansion should be carried out from fresh cord blood prior to long-term cryopreservation [31,32].

The impact of orthopaedic injuries to the health and revenue of the individual racehorse can be dramatic as was recently illustrated by the catastrophic and life-threatening injury that the 2006 Kentucky Derby champion "Barbaro" sustained during the 2006 Preakness Stakes. The financial magnitude of Thoroughbred racing in general is illustrated by the fact that in 2004 a total of 171 thousand Thoroughbred races were completed worldwide competing for a total purse value of 4 billion US dollars [1]. Bearing this in mind there is naturally a great interest in new possible treatment modalities for equine orthopaedic injuries which is also illustrated by the experimental application of autologous bone marrow MSCs and adipose tissue derived cell products to race and non-race horses with especially tendon problems [28-30]. The horse is a recognized animal model of OA induced injuries at various orthopaedic research facilities around the world for several reasons; the pathophysiology of OA appears similar between horses and humans, there are significant similarities in joint cartilage composition between horse and man and the shear size of horse joints and structures makes surgical manipulation easy compared to other domestic animal models [36]. The horse also provides a unique opportunity of studying naturally occurring injuries, which in many cases have considerable resemblance to injuries in human athletes. Deliveries of racehorses are generally observed making large-scale cord blood collection very feasible. For all of the above-mentioned similarities between horse and man, the horse now appears as a very favourable pre-clinical animal model for cord blood MSC research.

## Conclusion

This novel discovery of MSCs cells in equine cord blood and our preliminary insights into the differentiation potential of these cells could have significant future impact on the equine industry and individual horse health, but could also to generate significant biological, technical and procedural knowledge spillover and potentially proof-of-principle for future therapeutic uses of human cord blood derived USSCs or MSCs.

## Methods

### Cord blood collection

Cord blood was collected, without complications to the mare or foal, from seven foals immediately after foaling. The collection protocol was pre-approved by the Animal Care Committee at the University of Guelph (AUP 06R076). Cord blood was collected immediately after foaling and before the umbilical cord broke spontaneously or was broken according to farm management protocol. Venipuncture of the umbilical vein was performed with a 16 G hypodermic needle attached to a 450 ml blood transfusion collection bag (Fenwal) containing citrate phosphate dextrose adenine as the anticoagulant solution. The blood was then stored and transported at ambient temperature to the laboratory as quickly as possible.

### Mononuclear cell fraction isolation, seeding and MSC culture

The mononuclear cell fraction (MNCF) was isolated by carefully loading 30 ml of whole blood onto 10 ml of Ficoll density media (GE Healthcare Bio-Sciences) in 50 ml polypropylene tubes, centrifuge for 30 minutes at room temperature at 450 × *g *and the interphase collected after aspirating and discarding the supernatant. The interphase was washed with 20 ml PBS and centrifuged at 150 × *g *for 5 minutes at room temperature. The supernatant was aspirated and the cells were washed with PBS a second time. The cells were re-suspended in the isolation media and transferred to 6-well culture dishes that had been pre-treated with 100% FBS (Invitrogen) for 30–60 minutes to prevent adherence of monocytic cells as described by Bieback *et al*. (2004) [12]. The isolation media was low-glucose DMEM (Cambrex Bio Science) supplemented with 30% FBS, low dexamethazone (10^-7 ^M) (Sigma-Aldrich), penicillin (100 IU/ml) (Invitrogen), streptomycin (0.1 mg/ml) (Invitrogen), and ultraglutamine (2 mM) (Cambrex Bio-Science) as described by Kögler *et al*. (2004) [13]. Incubation was at 38.5°C in humidified atmosphere containing 5% CO_2_.

The isolation media was completely replaced after overnight incubation (12–18 hours) in order to remove non-adherent cells. Hereafter the media was completely replaced every 3 days until putative MSC colonies were noted. The cultures was inspected daily for formation of adherent spindle-shaped fibroblastoid cell colonies consistent with putative MSCs or contaminating osteo-like nodules and fungi. Initially expansion was done in 6-well culture dishes in order to allow pick-to-save procedures. Sub-culturing was performed at 60–80% confluence, when day-to-day colonic expansion was judged to slow down, or if signs of cellular detachment from the dish were noted in the center of the colony. Sub-culturing was done by chemical detachment using 0.04% trypsin/0.03%EDTA or by mechanically picking fibroblastoid colonies with a wiretrol pipette. Replating ratio after chemical cell detachment was 1:3. The cells in the second plate were referred to as passage 1 cells (P1). Dexamethasone was omitted from the expansion media, which was otherwise identical to the isolation media. Later, when cell numbers allowed, expansion was done in 25 cm^2 ^or 75 cm^2 ^tissue culture flasks. Cells were seeded at densities of 3000 – 25000 cells per cm^2^. Cells were cryopreserved after various passages numbers.

### Cryopreservation and thawing of MSCs

Cells were detached by 0.04% trypsin/0.03% EDTA (1 ml per 6–8 cm^2 ^culture surface area), washed in expansion media at 3 times the volume of trypsin/EDTA, spun down at 150 × *g *for 5 minutes, the supernatant was discarded and expansion media was added until the cell concentration was 1,000,000 – 2,000,000 cells per ml, then 1 ml of cold freezing solution, (DMEM high-glucose (Invitrogen) containing 10% FBS and 20% DMSO (Sigma-Aldrich)) was added per 1 ml of cell suspension and aliquots of 2 ml was stored overnight (12–18 hours) at -80°C in a isopropanol freezing canister. The next day the vials were plunged into liquid Nitrogen for long-term storage in liquid nitrogen.

Cryovials were thawed in a 37°C water bath for less than 3 minutes. The cells were immediately transferred to 5 ml of 37°C equilibrated expansion media and gently vortexed. The cells were centrifuged at 150 × *g *for 5 minutes at room temperature. The cell pellet was resuspended in a minimum volume of expansion media to allow a total cell count. Further expansion media was then added pending on the desired inoculum's cell concentration and volume.

#### *In vitro *differentiation studies

Osteogenic, chondrogenic and adipogenic differentiation potential has been evaluated for one of the three propagated cell lines isolated both before and after cryopreservation.

### Osteogenic differentiation

Undifferentiated cells were induced towards the osteogenic lineage using the protocol described by Jaiswal *et al*. (1997) [37]. Putative MSCs was seeded in six-well plates at a density of approximately 3000 cells/cm^2 ^and cultured in expansion media until reaching 90–100% confluence. Osteogenic differentiation was hereafter induced by culturing the cells for 20 days in osteogenic induction medium consisting of 100 nM dexamethasone, 10 mM β-glycerophosphate (Sigma-Aldrich), 0.05 mM L-ascorbic acid-2-phosphate (Fluka Biochemika), and 10% FBS in low glucose DMEM. As a negative control an equal number of wells were maintained in expansion media for 20 days. The media in both groups were completely replaced every 4 days. Osteogenesis was evaluated by colorimetric semi-quantitative assessment of calcium deposition and alkaline phosphatase activity as well as by Alizarin Red S and von Kossa histological staining.

### Semi-quantitative calcium deposition assay

Calcium deposition was evaluated according to the protocol described by Cambrex Bio Science [38]. All culture media was aspirated from each well and the wells were washed with 1 ml of PBS twice. After aspiration of the second PBS wash 0.5 ml of 0.5N HCl was added to each well. The cells were scraped of the well and transferred to a 1.5 ml Eppendorf tube. An additional 0.5 ml of 0.5NHCl was added to each well in order to wash of any remaining cells and this wash fluid was added to the Eppendorf tube. Eppendorf tubes with fresh samples were placed on an orbital shaker for 6 hours at 4°C. The Eppendorf tubes were then centrifuged at 500 × *g *for 2 minutes. The supernatant, containing the extracted calcium, was carefully aspirated without disrupting the pellet and transferred to another 1.5 ml Eppendorf tube. A standard curve was generated following the instruction provided in the Stanbio Laboratory Calcium (CPC) Liquicolor^® ^kit and the calcium concentration in control and osteo-induced samples were then determined. The calcium absorbance was measured at 570 nm by spectrometry.

### Semi-quantitative alkaline phosphatase enzyme activity assay

Cell lysate supernatant was evaluated for alkaline phosphatase activity. The culture wells were rinse twice with PBS for 3 minutes. Half a millilitre of PBS was added to the well and the cells were scraped off with a tissue scraper and transferred to a 1.5 ml Eppendorf tube. The wells were rinsed with 0.5 ml PBS, which was transferred to the Eppendorf tube as well. The sample was centrifuged at 5000 × *g *for 8 seconds. Maximum PBS was aspirated and the cell pellet was resuspended in 0.1 ml lysis buffer (500 ml lysis buffer: 250 mg sodium deoxycholate, 5 mg phenylmethyl sulfonyl fluoride (PMSF), 5 mg aprotinin, 500 ml nonidet P-40, 500 ml 10% (wt/vol) SDS) by pipetting the cells up and down several times. The tubes were left on ice for 5 minutes and vortexed for 30 seconds. The tubes were microcentrifuged at maximum speed for 10 minutes at 6°C. Fifty microliters of the supernatant was added in duplicate from each osteo-induced and control sample to wells in a 96-well plate. The kit, p-Nitrophenyl Phosphate Liquid Substrate System (Sigma-Aldrich), was used and 50 μl of p-nitrophenyl phosphate (pNPP) was added to each well. The absorbance was read at 405 nm on a microplate reader as soon as possible after adding the pNPP (time 0) and subsequent every 5 minutes for 20 minutes. The absorbance was read against 100 μl of undiluted pNPP. Covering it with aluminium foil screened the microplate from light between readings.

### Culture well total protein concentration

Both calcium deposition and alkaline phosphatase activity was evaluated against the total protein content of the culture wells. Total protein concentration was determined using a DC Bio-Rad protein method. In detail, 5 μl duplicate samples of the cell lysate supernatant were added to wells of a 96-well plate. Twenty-five microlitres of working reagent A (20 μl of reagent S per 1 ml of reagent A) and 200 μl of reagent B were added to each sample. A standard curve was generated from bovine serum albumin samples containing 4, 2, 0.5 and 0.25 mg/ml, respectively. The microplate was read at an absorbance of 630 nm after 15 minutes.

### Alizarin Red S staining

Alizarin Red S (Sigma-Aldrich), staining calcium deposits orange red, was done by carefully aspirating the medium from each well so as not to aspirate the cells. The cells were fixed incubating in ice-cold 70% ethanol for 5 minutes at room temperature, before carefully aspirating the alcohol and rinsing twice (5 minutes each) with water. The water was aspirated and 1 ml 2% Alizarin Red S solution was added. The plate was incubated at room temperature for 3 minutes, before removing the Alizarin Red S solution and washing the wells five times with 2 ml water. The pH value of the Alizarin Red S solution was adjusted to 4.1–4.3 with ammonium hydroxide prior to the procedure.

### Von Kossa staining

Von Kossa staining of calcium-phosphate deposits was performed. The cells were washed 3 times with PBS before fixation of the cells with 10% formalin for 1 hour at room temperature. The cells were washed 5 times with distilled water before adding 1 ml of 5% silver nitrate (Sigma-Aldrich) and exposing to UV light for 45 minutes. The wells were washed 5 times with distilled water. Sodium thiophosphate (5%) (Sigma-Aldrich) was added for 2 minutes and the cells were washed 3 times in distilled water. Nuclear fast red (Sigma-Aldrich) staining was done for 1 minute and the cells washed 5 times with distilled water. Calcium-phosphate deposits stained black, nuclei red and other tissues pink.

### Chondrogenic *in vitro *differentiation

Chondrogenic differentiation was performed using a micromass culture system [39]. Briefly, undifferentiated cells (2.5 × 10^5 ^cells) in a 15 ml polypropylene tube were centrifuged at 150 *g *for 5 min at room temperature to form a pellet. Without disturbing the pellet, the cells were cultured for 2 and 4 weeks in 0.5 ml complete chondrogenic differentiation medium (Cambrex Bio Science) containing 10 ng/ml TGF-β3. Media was completely exchanged every 3 days. Pellets were fixed in 10% formalin, imbedded in paraffin blocks and sectioned into 5 um sections. Hematoxylin and Eosin stain (Sigma-Aldrich) as well as Safranin O (Sigma-Aldrich) staining of glycosaminoglycans confirmed chondrogenic differentiation histologically. Hematoxylin and Eosin staining of slides were done in a routine manner. For the Safranin O staining, sections were stained with 0.1% aqueous Safranin O for 5 min and the nuclei was counterstained with Weigert's iron hematoxylin (Sigma-Aldrich) according to the protocol from Cambrex Bio Science [38].

### Adipogenic *in vitro *differentiation

Adipogenesis was induced using the protocol described by Pittenger *et al*. (1999) [40] and a slightly modified protocol of the one reported by Janderová *et al*. (2003) [35]. Six-well culture plates were seeded at 2.1 × 10^4 ^undifferentiated MSCs per cm^2 ^of tissue culture surface area in 0.3 ml of media per tissue culture surface. The cells were expanded until 100% confluency in regular expansion media. The expansion media was completely changed every 3 days.

At confluency some wells were stained to evaluated the baseline formation of neutral lipid-vacuoles stainable with Oil Red O (Sigma-Aldrich). The used Oil Red O staining protocol was as follows; aspirate all the media off the cells, wash once with 2 ml of PBS, replace the PBS with 2 ml of 10% formalin for 30 min at room temperature to fixate the cells, replace the formalin with 2 ml of sterile water for a few minutes, replace water with 60% isopropanol for 5 minutes, replace isopropanol with 2 ml of Oil Red O working solution made up as described by Cambrex, after 5 minutes the Oil red O solution was washed of indirectly with tap water. Two ml of Harris hematoxylin (Sigma-Aldrich) was added to the well for 1 minute before being aspirated and the wells washed with warm tap water. The wells were viewed using an inverted phase contrast microscope. Lipids appeared red and nuclei appeared blue.

The remaining plates were after the cells reached 100% confluency exposed to regular expansion media (negative control), adipogenic induction media alone, (1 μM dexamethasone, 0.5 mM 3-isobutyl-1-methyl-xanthine (IBMX), 10 μg/ml recombinant human (rh) insulin, 0.2 mM indomethacin, and 10% FCS in DMEM (Cambrex Bio Science)) adipogenic maintenance media alone (10 μg/ml rh insulin and 10% FCS in DMEM (Cambrex Bio Science)), cyclic exposure to adipogenic induction media for 72 hour followed by 24 hours of maintenance media, adipogenic induction media with 15% rabbit serum (Sigma-Aldrich) alone, or cyclic exposure to 72 hours of adipogenic induction media with 15% rabbit serum followed by 24 hours of maintenance media. The media was completely replaced every 3 days in wells not undergoing a cyclic protocol. Adipogenic potential was assessed by Oil Red O staining as described previously after 21 days of exposure to induction medias.

### Statistical analysis

Determination of any significant statistical difference between control and induced samples in calcium deposition and alkaline phosphatase activity was performed using SAS 9.0 software. The statistical method was ANOVA (analysis of variance) for a completely randomized design with sub-sampling. There were two treatment levels (control versus induced), three replications per level (3 culture wells per treatment) and two sub-samples per replication (duplicate measurements of absorbance values). The response was the absorption readings for alkaline phosphatase, calcium and protein measures.

## Authors' contributions

TGK participated in the study design, cord blood sampling, and all laboratory procedures and were the main contributor of the article draft. TH participated in cord blood sampling, most of the laboratory procedures and critiqued the final manuscript. PDT participated in the study design and critical revision of the manuscript. DHB participated in the study design, laboratory procedures, data analysis and critical revision of the manuscript.
